# P-534. Comparison of Concurrent (Same or Next-Day) Conventional Microbial Testing to Next Generation Sequencing (NGS) Results: Review of Pediatric Samples over 4-Years (2020-2024)

**DOI:** 10.1093/ofid/ofaf695.749

**Published:** 2026-01-11

**Authors:** Kathleen Condon, John Schieffelin, Margarita Silio

**Affiliations:** Tulane University School of Medicine, New Orleans, LA; Tulane University, New Orleans, Louisiana; Tulane University School of Medicine, New Orleans, LA

## Abstract

**Background:**

The role NGS has in diagnosis of infections remains unclear as high costs currently limit use. Strengths and limitations of this testing modality are still being studied.

**Figure:**
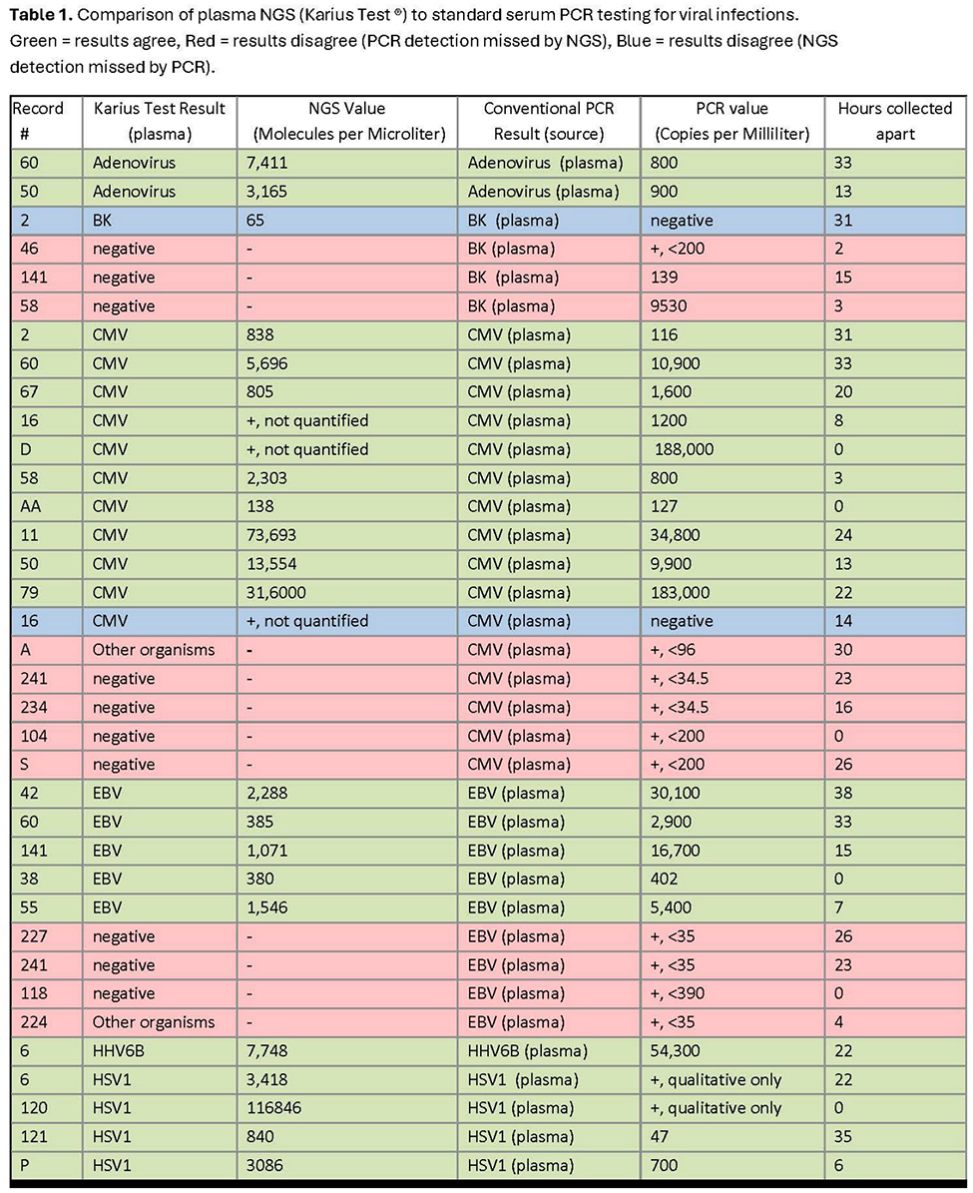

**Methods:**

271 plasma NGS (Karius Test ®) samples over 4 years were evaluated for the presence of conventional microbial testing occurring within one day. There were 68 instances for comparison (average 20.6 hours apart). Wound, non-BAL respiratory, stool and bacterial urine cultures were excluded. Positive NGS paired to negative blood culture were also excluded.

**Figure:**
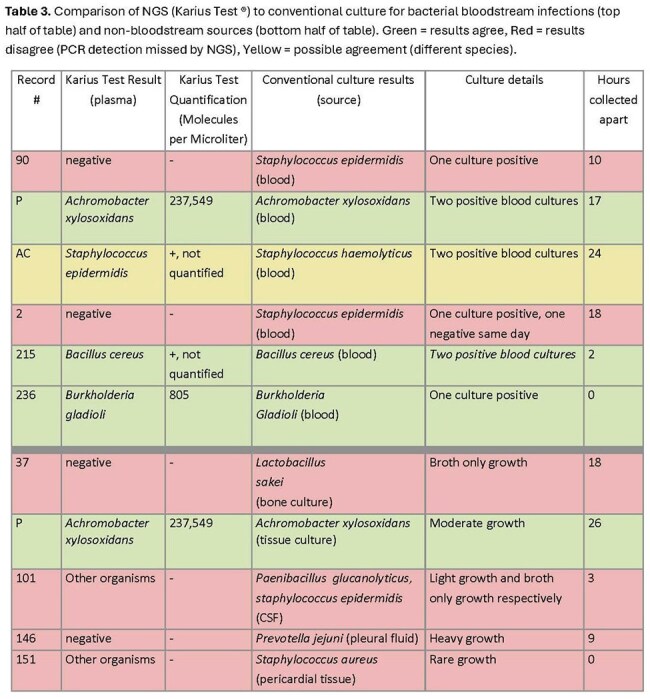

**Figure:**
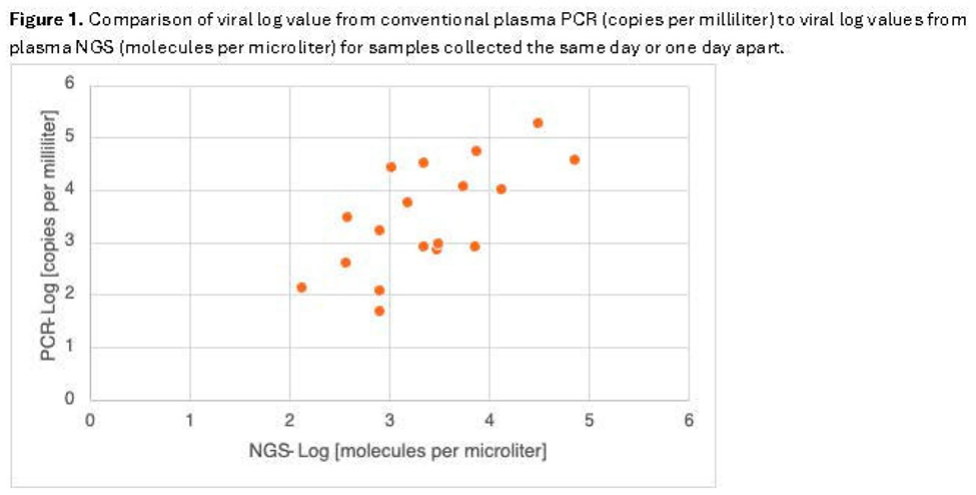

**Results:**

Viral: There was high concordance between NGS and viral plasma PCR (Table 1, Figure 1). When PCR viral load was above the threshold of quantification NGS successfully detected all cases of adenovirus, CMV, EBV, HSV1, and HHV6 (n=22). NGS detected CMV when PCR did not once. Viral PCR detection below the level of quantification (n=10) always corresponded to negative NGS. BK virus detection was variable. Twice PCR detected BK above quantification (139 and 9,530 copies/ml) but NGS did not. NGS detected BK once when it was undetected by PCR. Viral infections in other body compartments were inconsistently detected by NGS (Table 2).

Bacterial: All positive blood cultures corresponded to positive NGS except two cases of *staphylococcus epidermidis*, likely contaminants. NGS identified *Staphylococcus epidermidis* when *Staphylococcus haemolyticus* grew from culture. Most (5/6) cases of bacteria cultured from other body sites were missed by NGS although only two cases had greater than rare growth (Table 3).

Fungal: NGS did not detect dimorphic fungi in five instances of *Blastomyces* or *Histoplasma* antigen positivity. NGS failed to detect *Cryptococcus neoformans* fungemia and *Rhizopus* with dermal angioinvasion. NGS identified invasive fungal infections unable to be cultured twice (*Rhizopus, Aspergillus*).

**Conclusion:**

Plasma NGS detected viruses when above the threshold for quantification but not when below the limit of quantification. BK virus had variable detection. NGS identified bacteremia well. A positive blood culture with negative NGS suggests contamination and favors antibiotic de-escalation. Detection of bacterial infections outside the bloodstream is unreliable. Dimorphic fungi and *Cryptococcus* were not detected by NGS, while *Candida* and molds were.

**Disclosures:**

All Authors: No reported disclosures

